# The immunome of mobilized peripheral blood stem cells is predictive of long-term outcomes and therapy-related myeloid neoplasms in patients with multiple myeloma undergoing autologous stem cell transplant

**DOI:** 10.1038/s41408-023-00920-9

**Published:** 2023-09-26

**Authors:** Saurabh Zanwar, Eapen K. Jacob, Carl Greiner, Kevin Pavelko, Michael Strausbauch, Emilie Anderson, Arini Arsana, Megan Weivoda, Mithun Vinod Shah, Taxiarchis Kourelis

**Affiliations:** 1 Division of Hematology, Mayo Clinic, Rochester, MN USA; 2 Division of Transfusion Medicine, Human Cellular Therapy Laboratory, Rochester, MN USA; 3 Immune Monitoring Core, Mayo Clinic, Rochester, MN USA; 4 Division of Hematology Research, Mayo Clinic, Rochester, MN USA

**Keywords:** Diseases, Medical research

## Abstract

Upfront autologous stem cell transplant (ASCT) is the standard of care for newly diagnosed multiple myeloma (MM) patients. However, relapse is ubiquitous and therapy-related myeloid neoplasms (t-MN) post-ASCT are commonly associated with poor outcomes. We hypothesized that the enrichment of abnormal myeloid progenitors and immune effector cells (IEC) in the peripheral blood stem cells (PBSCs) is associated with a higher risk of relapse and/or development of t-MN. We performed a comprehensive myeloid and lymphoid immunophenotyping on PBSCs from 54 patients with MM who underwent ASCT. Median progression-free (PFS), myeloid neoplasm-free (MNFS), and overall survival (OS) from ASCT were 49.6 months (95% CI: 39.5-Not Reached), 59.7 months (95% CI: 55–74), and 75.6 months (95% CI: 62–105), respectively. Abnormal expression of CD7 and HLA-DR on the myeloid progenitor cells was associated with an inferior PFS, MNFS, and OS. Similarly, enrichment of terminally differentiated (CD27/CD28^-^, CD57/KLRG1^+^) and exhausted (TIGIT/PD-1^+^) T-cells, and inhibitory NK-T like (CD159a^+^/CD56^+^) T-cells was associated with inferior PFS, MNFS, and OS post-transplant. Our observation of abnormal myeloid and IEC phenotype being present even before ASCT and maintenance therapy suggests an early predisposition to t-MN and inferior outcomes for MM, and has the potential to guide sequencing of future treatment modalities.

## Introduction

Autologous stem cell transplantation (ASCT) and maintenance form the treatment backbone for most patients with multiple myeloma (MM) [1]. Along with the incorporation of novel immune-based treatment strategies, this paradigm has led to a consistent improvement in the outcomes of patients over the past two decades [2]. Despite this, a cure for MM remains elusive and most patients relapse. In addition, 2–4% of patients develop therapy-related myeloid neoplasms (t-MN) which are associated with a dismal survival, significantly contributing to post-ASCT mortality [3–5]. Thus, relapse and t-MN represent major barriers to improve the post-ASCT outcomes in MM. Therapy-related myeloid neoplasms are highly enriched in pathogenic variants in the tumor suppressor gene *TP53* (*TP53*^mut^), which explains their aggressive course and the lack of response to the currently available therapies [6]. There is emerging concern that chimeric antigen receptor T cell therapy (CAR-T) can be complicated by the development of t-MN in upto 10–20% of patients [7, 8].

Given that ASCT is associated with significant morbidity and resource utilization, there is a substantial interest in identifying high-risk patients for relapse and t-MN before ASCT. While the focus thus far has been the neoplastic cells, recently the role of tumor immunosurveillance has emerged. A permissive immune tumor microenvironment (iTME) contributes to treatment resistance and progression of MM [9, 10]. Increased T-cell exhaustion and immune senescence have been associated with poor outcomes after standard MM therapies, ASCT and novel T cell redirecting therapies [11–16]. Immune reconstitution following autologous [17] and allogeneic SCT [18] impacts the post-transplant outcomes and immunomodulation is being pursued to improve long-term outcomes.

Similarly, *TP53*^mut^ MN are characterized by a highly immunosuppressed immune milieu associated with a reduced number of immune effector cells (IEC) including cytotoxic T- and natural killer (NK)-cells, as well as the expansion of regulatory T-cells (Tregs), myeloid-derived suppressor cells (MDSC), and exhausted T-cells [19–22]. The expression of various exhaustion receptors is increased following hypomethylating agents and some patients with myelodysplastic syndromes can respond to checkpoint blockade [21, 22]. Finally, clonal expansion of terminally differentiated, immune senescent T-cells is seen in all MDS subgroups indicative of an ineffective T-cell response that fails to control disease progression [23, 24]. Peripherally mobilized blood-derived stem cell (PBSC) grafts are responsible for the reconstitution of the myeloid and immune compartments following the myeloablative doses of melphalan used in ASCT. We hypothesized that pre-existing immune abnormalities in the mobilized PBSC will lead to reconstitution of an immunosupressive iTME and contribute to early relapse and/or t-MN development. To that end, we explored the immune composition of mobilized PBSC and its relationship with subsequent MM relapse and t-MN development.

## Methods

### Study population

After institutional review board approval, we screened patients with active MM that were evaluated at Mayo Clinic, Rochester, MN between 01/01/2003 and 12/31/2020. Patients who underwent an ASCT and had cryopreserved PBSC products available for research were included. We enriched this cohort with patients who developed t-MN to study this group. t-MN was defined using the 2016 World Health Organization criteria [25]. The revised international staging system (R-ISS) was used to risk stratify MM patients [26]. Hematologic response to therapy and engraftment syndrome were defined per internationally accepted criteria [27, 28]. Short duration of remission for MM following ASCT was defined as <24 months if no post-ASCT maintenance was utilized or <48 months if post-ASCT maintenance was not utilized.

### Mass cytometry

The antibody panels included 37 lymphoid- and myeloid-based markers each (Supplementary Table 1). For primary conjugations, purified antibodies were obtained in carrier protein-free phosphate-buffered saline and labeled using the X8 polymer MaxPAR antibody conjugation kit (Fluidigm) according to the manufacturer’s protocol. All antibodies were titrated to optimal staining concentrations using peripheral blood mononuclear cells. Antibody master mixes were prepared fresh for each experiment. All samples were processed identically. PBSC were collected after mobilization using peripheral blood leukapheresis. Using validated processes in a Current Good Manufacturing Practice (cGMP) laboratory, the cells were concentrated and mixed with a cryopreservation solution to give a final white blood cell concentration of 300 × 10^6^ nucleated cells/ml in 10% DMSO, 10% plasma, and 30% PlasmaLyte-A (Baxter, Deerfield, IL) or Normosol-R (Hospira, Lake Forest, IL). The cells were then frozen using a controlled rate freezing process and stored in vapor phase liquid nitrogen tanks. The main products were stored in cryobags ranging from 50–100 ml in addition to 1.5 ml cryovials for additional testing as needed. Cryopreserved cells were resuscitated for mass cytometry analyses by rapid thawing and were rested in RPMI 1640 (20% fetal bovine serum) for 60 min prior to staining. Staining was performed using Fluidigm’s protocol. Briefly, 1–3 million cells were stained for viability with 5 mM cisplatin for 5 min at room temperature and quenched with cell staining medium (CSM; Fluidigm). Cells were then incubated for 10 min at room temperature with human FcR blocking reagent (Biolegend) and stained with the surface antibody cocktail for 60 min at 4 °C with gentle agitation. Finally, cells were washed twice with CSM, fixed with 1.6% paraformaldehyde, washed with CSM, and resuspended in 1:1000 solution of Iridium intercalator diluted in MaxPar Fix and Perm buffer (Fluidigm) for 20 min at room temperature. Prior to the acquisition, cells were washed twice in CSM and twice in deionized water and were then diluted to a concentration of 0.5 million cells per milliliter in water containing 10% of EQ 4 Element Beads (Fluidigm). Cells were filtered through a 35-μm membrane prior to mass cytometry acquisition. Samples were then acquired on a Helios mass cytometer.

### Mass cytometry data analysis

Flow cytometry standard files were normalized and concatenated using the Fluidigm acquisition software. Flow cytometry standard files were uploaded to the OMIQ software from Dotmatics (www.omiq.ai, www.dotmatics.com), where transformation and cleaning (doublets, debris), were done as previously described [11]. To correct for between-sample technical variations (“batch effects”), we used fdaNorm [29] within the omiq.ai platform. Characteristic images of marker expression before and after normalization are shown in Supplementary Fig. 1. Clustering and visualization were performed within the omiq.ai platform using PhenoGraph [30] and UMAP [31], respectively. When clustering, 50,000 CD45+ events per file were used when using the lymphoid panel due to computational constraints. When using the myeloid panel, we manually excluded T-, B-, and NK-cells using canonical markers (CD3, CD19, and a combination of CD7 and CD56, respectively) and clustered the remaining cells and using 50,000 CD45+ events per file. Identified clusters were then exported for downstream statistical analyses. All immune subset frequencies are reported as a percent of CD45+ cells. All raw mass cytometry data are uploaded to FlowRepository (https://flowrepository.org/).

### Statistical methods

Fisher’s exact test was used to compare categorical variables and Wilcoxon rank sum (2 groups) or Kruskal-Wallis test ( > 2 groups) for continuous variables. Kaplan-Meier survival analysis was used to estimate the overall survival (OS) from diagnosis of MM, and progression-free survival (PFS) and tMN-free survival (MNFS) from ASCT. The log-rank test was used to compare groups. A Cox proportional hazards model was used to perform multivariable analyses for time to event outcomes. Patients alive and without progression to MM or t-MN, were censored for PFS and MNFS analyses, respectively. Hierarchical clustering of patients according to the abundance of immune subsets was performed using Ward’s minimum variance method. Principal component analyses were performed using log transformed and scaled values of immune subset frequencies. Statistical analyses were performed using the JMP Pro statistical software version 14.1 (SAS Institute, Cary, NC). Correlation analyses were performed in R using the corrplot package. A 2-sided false discovery rate (FDR) adjusted *P*-value of <0.05 was considered significant when multiple comparisons were performed; otherwise, a *P*-value of <0.05 was considered significant.

## Results

The baseline clinical characteristics of the 54 patients included in the study are shown in Table 1. Given the referral nature of our institution, patients with high-risk diseases were overrepresented. Also, given the limitations imposed by sample availability, we included patients that had PBSCs collected but did not receive lenalidomide maintenance, which became standard of care later in the study time frame. As a result, 19 patients did not receive post ASCT maintenance.

**Table 1:** Baseline Characteristics for patients in the study.

Parameter at Diagnosis of MM	*N* = 54 (%)
Age in years, median (range)	61.3 (32.7–73.3)
Sex (% females)	16 (30%)
ISS stage 3 (%)	15(28%)
LDH > ULN (%)	6 (11%)
High Risk Cytogenetics, *n* (%)*	22 (41%)
t(4;14)	4 (8%)
Deletion 17p	3 (6%)
1q duplication	13 (24%)
t(11;14)	10 (18%)
t(14;16)	2 (4%)
Hyperdiploidy	24 (46%)
Deletion 13q	13 (24%)
R-ISS stage 3	18 (34%)
Bone marrow plasma cell infiltrate, median % (range)	40
**ASCT data**	
Time from MM Diagnosis to ASCT in months, median (range)	6.3 (2.5–51)
**Induction therapy prior to ASCT**, *n* (%)	
Doublet induction (%)	11 (20%)
Triplet induction (%)	43 (80%)
**Mobilization strategy** (%)	
G-CSF alone	25 (46%)
G-CSF+ plerixafor	24 (45%)
G-CSF+ cyclophosphamide	4 (7%)
G-CSF + plerixafor + cyclophosphamide	1 (2%)
**Conditioning for Transplant** (%)	
Melphalan 200 mg/m^2^	40 (74%)
Melphalan 140 mg/m^2^	10 (19%)
Melphalan 170 mg/m^2^	2 (4%)
Melphalan+TBI	2 (4%)
**Transplant time period** (%)	
2001–2005	2 (4%)
2006–2010	4 (8%)
2011–2015	28 (52%)
2015–2020	20 (37%)
Median Time to engraftment, days	18
Severe Engraftment Syndrome, (%)	7.4
**t-MN Data**	
t-MN after ASCT, *n* (%)	20 (37%)
Time from ASCT to t-MN in years, median (range)	4.9 (1.7–11.8)
*TP53* mutation at tMN, n (%)	7 (39%)
Lines of treatment before t-MN, median (range)^#^	2 (1–9)
Short Responders, *n* (%)	8 (40%)
**Maintenance therapy after ASCT**	*N* = 35
Lenalidomide maintenance^¥^	92% (32 out of 35)
Duration of Lenalidomide maintenance, median (range) years	1.6 (0.3–9)

*ASCT* autologous stem cell transplantation, *ISS* international staging system, *G-CSF* granulocyte colony stimulating factor, *MM* multiple myeloma, *TBI* total body irradiation, *tMN* therapy related myeloid neoplasm.

*high risk cytogenetics per mSMART 3.0: deletion 17p, t(4;14), 1q duplication, t(14;16)/t(14;20); ^#^Lines of therapy prior to the development of tMN in the tMN cohort versus lines of therapy till last follow-up for patients without tMN; ^¥^2 patients received bortezomib and 1 patient received ixazomib as maintenance therapy. All percentages are rounded off.

### The immune landscape of mobilized PBSC

**Fig. 1:**
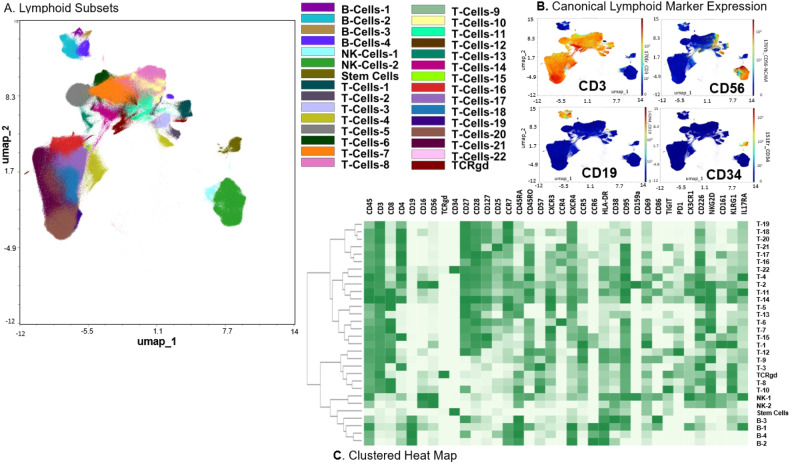
Lymphoid Subsets. **A** Phenograph Clusters projected on UMAP plot of all identified lymphoid subsets (NK, T, and B cells) and CD34+ cells using the lymphoid panel. Myeloid or lineage negative subsets were excluded for clarity. **B** Canonical marker expression (CD3, CD19, CD56, CD34) of major lineages. **C** Clustered heatmap showing the expression of all markers (lymphoid panel) expressed in identified lymphoid subsets across all files.

**Fig. 2:**
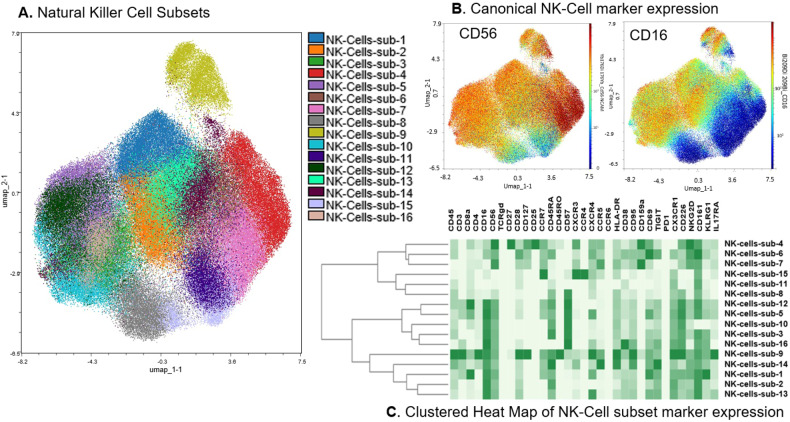
Natural Killer Cell Subsets. **A** Phenograph clusters projected on UMAP plot of all identified NK cell subsets after sub-clustering manually gated NK Cells and using the lymphoid panel. **B** Canonical NK-cell marker expression (CD16, CD56). **C** Clustered heatmap showing the expression of all markers expressed in NK cell subsets after sub-clustering of manually gated NK cells.

**Fig. 3:**
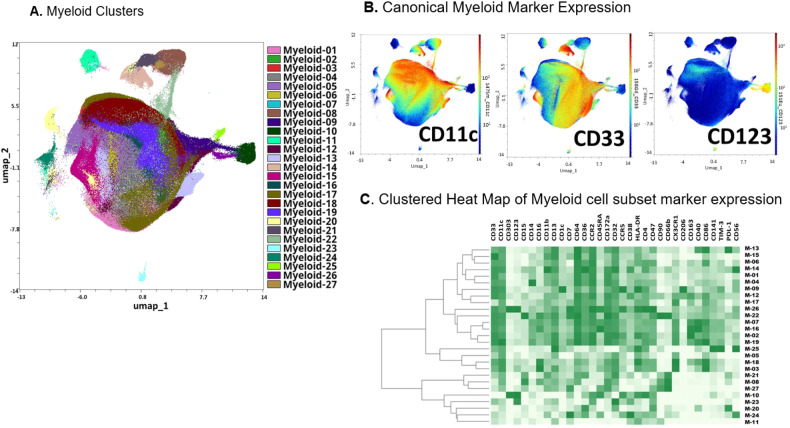
Myeloid Subsets. **A** Phenograph Clusters projected on UMAP output of all identified myeloid subsets identified by clustering all CD45+ non-T, B, or NK cells. Lineage-negative clusters not expressing canonical myeloid markers (CD11c, CD33) were excluded. **B** Canonical marker expression (CD33, CD11c, CD123). **C** Clustered heatmap showing the expression of all markers expressed in myeloid cell subsets after sub-clustering of manually gated myeloid cells.

We generated 2-dimensional UMAP maps of the data generated by the 2 panels (lymphoid and myeloid) in the manually gated CD45+ cells (Supplementary Figs. 2 and 3, respectively). These analyses demonstrated that major lineages separated well in the 2-dimensional space. The most abundant cell populations were T-cells (49% of the CD45+ cells) and myeloid cells (43% of the CD45+ cells, Supplementary Fig. 4), which was not surprising considering these were mobilized cells. We then clustered all CD45+ cells using the lymphoid panel and projected the identified clusters on the respective UMAP (Fig. 1A, B). A heatmap of the marker expression of the lymphoid populations identified with the lymphoid panel is shown in Fig. 1C. To better characterize T- and NK-cell diversity, we subclustered on the manually gated respective cell subsets and excluded the markers not expressed on T- and NK-cells, respectively. Subclustering on T-cells did not increase the T-cell cluster diversity (not shown). Therefore, only the T-cell clusters that resulted after clustering with the lymphoid panel on CD45+ cells were considered for further analyses. When subclustering on NK-cells however, an additional 16 NK-cell clusters were identified and were projected on a 2-dimensional UMAP map (Fig. 2A, B). The corresponding heatmap of their marker expression is shown in Fig. 2C. We then subclustered on manually gated myeloid cells (excluding markers not expressed on myeloid cells) and identified 27 myeloid clusters which were projected on a UMAP map (Fig. 3A, B). A heatmap of their marker expression is shown in Fig. 3C.

Next, we explored the associations between the immune subsets and the dichotomized clinical outcomes of interest. To identify if specific immune subsets correlated with short or long remissions, the frequencies of the identified immune subsets between the 2 groups were compared. We found no differences at our prespecified level of significance (FDR corrected *P*-value < 0.05). When considering trends (non-FDR corrected *P*-value < 0.05), the activated (CD69/CD161/KLRG1^+^) memory (CD27/CD28/CD127^+^) CD8 T-1 cell subset was enriched in the patients with long remission compared to those with short remission (0.9% *vs*. 0.3%, *P* = 0.02). Similarly, no significant differences between the patients who subsequently developed t-MN compared to those who did not were identified. Several trends of populations of potential interest became apparent (Supplementary Table 2). NK-cell subcluster-7, characterized by CD56^high^, CD159a^+^ (NKG2A) phenotype, was enriched in the patients who subsequently developed t-MN (0.33% *vs*. 0.17%, *P* = 0.006). Enrichment of these well-described inhibitory NKG2A^+^ NK-cells has been noted in the bone marrow of patients with MDS and AML [32]. A “terminally mature” [33, 34] CD56/CD16/CD27^dim^, CCR4/CXCR3/CCR7^high^ NK-15 subset was enriched in patients developing t-MN (0.09% *vs*. 0.06%, *P* = 0.001), and is thought to have T-cell immunosuppressive properties [35]. The T-cell subsets T-5 and T-20, two CD8+ naive subsets with bone marrow homing (CXCR4 + ) [36] and cytotoxic (NKG2D + , CD226 + , respectively) receptors were decreased in patients who later developed tMN as was the T-1 T cell subset described above. These observations imply that changes associated with decreased immunosurveillance may be evident in G-CSF mobilized PBSC products years before the development of t-MN.

We then compared patients that had been mobilized with G-CSF alone or G-CSF and plerixafor and found no differences (including those with a non-FDR corrected *p*-value < 0.05), suggesting that plerixafor use does not significantly influence the immune contexture of mobilized PBSC.

### The structure of the immune landscape correlates with clinical outcomes

**Fig. 4:**
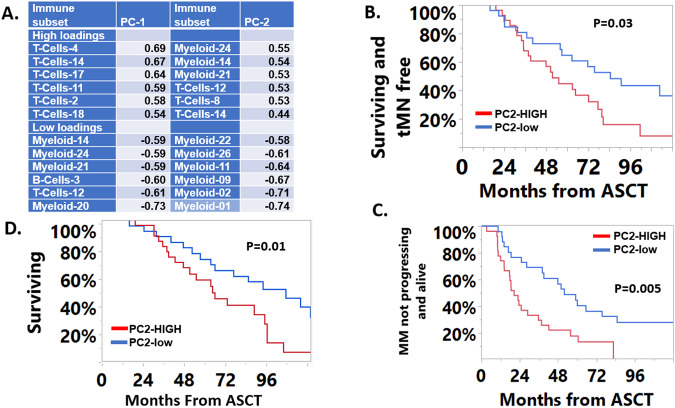
Correlation of Immune subsets with Clinical Outcomes. **A** Immune subsets with the highest/lowest loadings for each principal component (PC). **B** Patients with high PC-2 scores had worse tMN-free survival, **C** progression-free survival from transplant and **D** inferior overall survival from ASCT.

We hypothesized that the immune subsets with highly correlated frequencies were more likely to be co-regulated. A correlation matrix and a list of all identified significant (*P* < 0.05) correlations sorted by their R values are shown in Supplementary Fig. 5 and provided as a Supplemental spreadsheet, respectively. This analysis identified 3 patients with unique immune compositions and poor outcomes (supplemental information). To further explore the major drivers of variability in the data, we performed a principal component (PC) analysis. The first 2 PCs (PC-1 and PC-2) explained 15.2% and 12.5% of the variability in the data, respectively. The top and bottom 10% of immune subsets with the highest and lowest loadings for each PC are shown in Fig. 4A. Conceptually, an immune subset with high loadings within a given PC, correlates the most with that PC and is most influential in defining its immune subset composition. Consequently, patients that have high scores for this PC are expected to have higher abundance of these immune subsets. Immune subsets with high loadings within PC-1 consisted exclusively of CD4^+^ cells with early memory phenotypes (CD27/CD28/CD127^+^), whereas those with low loadings for PC-1 consisted mostly of the rare myeloid and T cell subsets that were identified during the correlation analyses described above and overlapped with most subsets with high loadings within PC-2. Finally, immune subsets with the lowest loadings for PC-2 consisted exclusively of myeloid subsets, some of which with phenotypes consistent with plasmacytoid dendritic cells (pDCs; M-26:CD123/CD303 + ) or progenitor cells (M-22, M-11: CD13/CD90/CD7/CD15 + ). These data suggest that the major drivers of variability in PBSC include subsets associated with an improved or impaired immunosurveillance (e.g., early memory CD4 subsets), various myeloid subsets, and as expected, mobilized progenitor cells.

Next, we analyzed these components to explore the relationships between immune system composition with clinical outcomes of interest. A high PC-2 score was associated with an inferior OS and PFS from ASCT and MNFS (Fig. 4B–D). Worse OS was also noted from MM diagnosis (not shown). Interestingly, despite the difference in outcomes, the baseline characteristics were largely comparable between the two cohorts, except for age and R-ISS stage 3 status. Patients with high PC-2 scores were older (63 *vs*. 59 years, *P* = 0.009) and were noted to have higher proportion of R-ISS stage 3 disease (54% *vs*. 14%, *P* = 0.005). In a multivariate analysis that include PC-2 score status (high *vs*. low), R-ISS stage 3 disease and age, PC-2 score status was not independently associated with PFS and OS from ASCT (not shown). In contrast, the high PC-2 cohort was associated with an inferior MNFS (Fig. 4B). The high PC-2 score remained an independent predictor of shorter MNFS on a multivariate analysis [HR 2.2 (95%CI: 1.1–4.4), *P* = 0.036] including lines of therapy received and whether lenalidomide maintenance was used or not. To understand the relationship between MNFS and MM PFS, we evaluated the extent of overlap between the development of tMN and progression of MM. Sixteen patients did not have MM progression during follow-up, of these 7 developed tMN. Thirty-eight patients had MM progression during follow-up, of which 13 also developed tMN. In these 13 cases, 12 patients developed tMN much later than the first MM progression with the exception of one patient who was diagnosed with tMN at the same time as their first MM relapse.

**Fig. 5:**
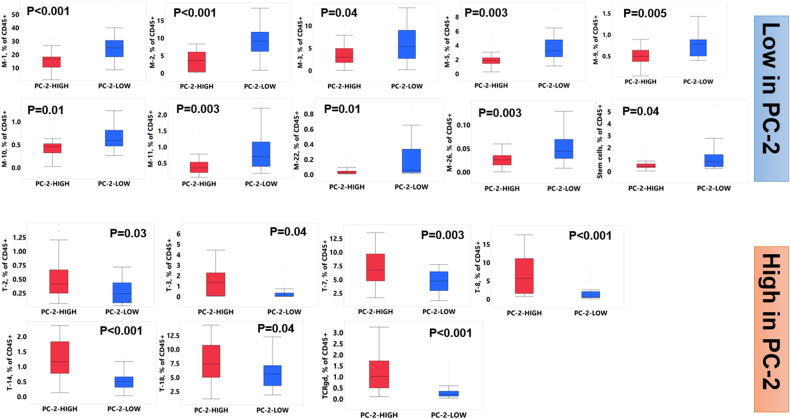
Immune subsets significantly different (FDR p value < 0.05) between patients with high (PC-2-High) and low (PC-2-Low) PC-2 scores. All p-values noted are FDR corrected.

Immune subsets that were significantly different (FDR corrected *P*-value < 0.05) between the patients with high and low PC-2 scores are shown in Fig. 5. These, not unexpectedly, largely overlap with the dominant (high/low loadings) immune subsets within PC-2. Immune subsets enriched in the high PC-2 cohort included the “NKT-like” (CD56 + ) T-2 subset which expressed the inhibitory CD159a receptor [37] and TIGIT; the terminally differentiated (CD27/CD28-, CD57/KLRG1 + ) and exhausted (TIGIT/PD-1 + ) T-3 subset the exhausted (TIGIT/PD-1 + ) T-7 and T-14 subset, the immunosenescent (CD27/CD28-, CD57/KLRG1 + ) T8 subset as well as the terminally exhausted (C27/C28-, KLRG1/PD-1/TIGIT + ) TCRgd subset. Among subsets that were low within the patients with high scores for PC-2 included the HLA-DR expressing M2 subset; the CX3CR1 + M-3 subset that expressed markers of nonclassical monocytes (CD14dim/CD16 + ) and M1 polarization (CD86/CD16 +); the M-9, M-10 and M-26 subsets that expressed dendritic cell markers (CD1c/HLA-DR+ and CD123/CD303/HLA-DR + , respectively), the M-11 and M-22 subsets with a phenotype consistent with progenitor cells (CD90/CD7/CD13 + ). Given the known detrimental effects of malignant plasma cells on BM hematopoiesis [38, 39], and T cell immunosenescense [14] which could be reflected in the abundance of these subsets in PBSC, we evaluated differences in the quality of hematologic response after induction chemotherapy (VGPR or better versus not and degree of clearance of bone marrow plasma cells) between PC-2 high and PC-low patients but found no differences. These data suggest that T cell subsets associated with decreased tumor immunosurveillance are increased, whereas those associated with antigen presentation (DCs, HLA-DR + ) improved tumor outcomes (M1 monocytes) and progenitor cells are decreased in the PBSC products of patients with poor outcomes after ASCT.

## Discussion

The utility of ASCT, a universally utilized modality in MM, is hampered by two long-term complications: relapsed MM and the development of t-MN. ASCT is often associated with high morbidity, financial toxicity, and high resource utilization. Therefore, the question of the continued relevance and timing is often raised [40]. Improved identification of patients who are likely to derive lesser benefit and/or develop t-MN may improve ASCT outcomes, while minimizing the toxicities. Given the impact of PBSCs in post-ASCT immune reconstitution, we hypothesized that the immune composition of the PBSCs can identify patients with worse outcomes.

In this study, we provide, for the first time, a comprehensive immune atlas of the lymphoid and myeloid compartment of PBSC of patients undergoing upfront ASCT for MM. We find that PBSC products enriched in immunosuppressive T and myeloid cell subsets and with a lower proportion of antigen-presenting cells, progenitor populations, and anti-tumor macrophages are associated with shorter PFS and MNFS. Specifically, the enrichment of senescent and exhausted T-cells was associated with inferior PFS/MNFS. T-cell senescence and exhaustion are known to be associated with inferior outcomes in MM at various disease stages, including at diagnosis and following ASCT [11, 17, 41]. Various factors can impact T-cell exhaustion, including chronic tumor-associated antigenic stimulation, secretion of immune suppressive cytokines by the iTME and as an effect of treatment for the malignancy. MM patients exposed to more lines of therapy develop a progressively dysfunctional iTME with relapsed/refractory MM having an abundance of senescent T-cells and decreased early memory T-cells [42]. These findings carry potential significance beyond ASCT, as these immune effector cell subsets are associated with inferior responses with subsequent T-cell redirecting therapies including bispecific antibodies and CAR-T cells [43]. Our findings suggest that these dysfunctional T-cells may arise early in the disease course and predict future inferior outcomes.

We also show that the patients with a higher proportion of exhausted T-cells had a shorter MNFS. Patients with de novo AML have high expression of inhibitory molecules (PD-1, TIGIT) on CD8 + T-cells, which in turn are associated with therapeutic resistance and disease progression [44, 45]. This immune exhausted phenotype is even more prominent in *TP53*^*mut*^ myeloid neoplasms, which can upregulate PD-L1 expression in hematopoietic stem cells and increase the proportion of highly immunosuppressive regulatory T-Cells [19].

We observed enrichment of several differences NK-cell subsets in patients who subsequently developed a t-MN. Specifically, subsets of CD56+ expressing NK- and T-cells with the co-expression of the inhibitory receptor CD159a (NKG2A) were enriched in patients with inferior outcomes. Impaired NK-cell distribution with a phenotypic shift from a mature to immature state along with an impaired NK antitumor response in relation to the inhibitory CD159a expression has been previously demonstrated in patients with MDS and AML. The CD159a is an inhibitory immune checkpoint expressed on cytotoxic T-cells and NK-cells and upregulated in immune microenvironment of tumors treated with PD-L1 inhibitors, suggesting this to be a possible escape mechanism [46, 47]. Blockade of CD159a with targeted monoclonal antibodies has been shown to augment anti-tumor immunity as well as response to cancer vaccines and blocking CD159a may be a strategy worth exploring in patients with MM as well [48, 49]. Additionally, NK-cells with a low CD159a expression may have a role in aging-associated immune surveillance and CD159a has been shown to be upregulated in patients with ineffective erythropoiesis and MDS [37]. This may further explain the higher risk of tMN in patients enriched with CD159a expressing NKT-cells in our cohort.

Apart from being enriched in dysfunctional T-cell subsets, we show that patients with inferior MM-related outcomes and shorter tMN-free survival also had lower proportion of myeloid cells with antigen-presenting properties (HLA-DR + , pDC phenotype). Studies in precursor MM states (including MGUS and smoldering MM) have demonstrated impaired HLA-DR expression on CD14+ monocytes, which was noted to promote in vitro myeloma cell growth and suppress T-cell activation [50]. This may explain the worse MM-related survival noted in patients with lower proportion of HLA-DR expressing myeloid subsets [50]. We were able to validate the abnormal expression of CD7 as well as HLA-DR to be associated with increased t-MN risk. In patients undergoing ASCT, the presence of CD7 was associated with 6.6-fold higher risk t-MN risk [51]. Similarly, abnormal expression of CD7 and HLA-DR/CD13 was associated with 3.5- and 2.9-fold higher risk of a subsequent MN in patients with clonal cytopenia of undetermined significance (CCUS) [52].

Our findings carry implications for future therapies including CAR-T and bispecific T-cell engagers. There is ample evidence of the limited durability of the efficacy of CAR-T therapies due to the development of T-cell exhaustion and senescence [53, 54]. Pre-existing IEC dysfunction, therefore, may provide a window into assessing appropriate candidates for CAR-T therapy and further impress upon the need for developing ‘exhaustion resistant’ CARs [55, 56]. Finally, the development of t-MN after CAR-T therapy is an area of increasing concern and it is important to be able to identify patients that might have a predisposition to t-MN while assessing for CAR-T therapy. Limited data for post CAR-T t-MN suggests a somewhat overlapping transcriptional profile for t-MN developing after CAR-T therapy with de novo AML/MDS [57]. Finally, our results suggest that utilizing PBSC products as a source for CAR-T may not bypass the problem of immune dysfunction inherent in this patient population. Other sources (e.g., allogeneic CAR T) may address this issue better.

ASCT and post-ASCT lenalidomide exposure are associated with approximately 100- and 6-fold increased risk of t-MN [4, 58]. Our study suggests that the difference in immunome predates ASCT and post-ASCT lenalidomide exposure and provides a novel insight into t-MN pathogenesis. Previously, the pathogenesis of t-MN was considered a unidimensional phenomenon characterized by therapy-induced DNA damage that led to leukemic transformation of a HSC. Recently, a more nuanced model has emerged: a preleukemic clone may exist before the exposure to DNA-damaging therapies [59] and persist thereafter without manifesting as t-MN [60]. In addition, the role of non-HSC factors such as mesenchymal stromal cells has been described [61].

Factors that result in ‘tolerance’ of the clone versus progression into an overt leukemia are yet not known. Our observations suggests that the risk of t-MN predates the ASCT and maintenance therapy and point towards the importance of immune surveillance in predisposing to the development of t-MN. Conversely, the pre-existence of such anomalies paves the way for early identification, prevention, and sequencing of the available therapeutic strategies. For example, patients with such PBSC immunome could be considered for a deferred ASCT, shorter exposure to or avoidance of lenalidomide.

Limitations of our study include the selection of the study population based on the available samples rather than a pre-defined criterion. Disproportionate loss of some myeloid immune subsets remains a possibility when using cryopreserved cells. The small sample size limits the generalizability of these data which need additional validation. The immune subsets were not independently prognostic when adjusted for age and R-ISS stage. Additionally, since these phenotypes were also associated with higher R-ISS stage they may reflect a negative impact of a more aggressive tumor itself on the IEC pool [14, 62]. Finally, it is unclear if the changes noted in PBSC are also present in peripheral blood samples that would be easier to obtain. We chose to investigate PBSC since it represents the source of bone marrow engraftment after myeloablative conditioning.

Despite these limitations, our findings of the PBSC immunome composition predicting inferior MM-related outcomes and future risk of tMN will guide future confirmatory studies. The use of a wide array of therapies and the protracted follow-up needed make such studies challenging. Therefore, our study is uniquely positioned to answer the question of the impact of the PBSC immunome on future risk of relapse and tMN development.

### Supplementary information

Correlation Matrix Supplementary Sheet

Supplementary Material

## Data Availability

The raw data from the mass cytometry experiments are available at www.flowrepository.org.
