# Supplementary material for: The immunome of mobilized peripheral blood stem cells is predictive of long-term outcomes and therapy-related myeloid neoplasms in patients with multiple myeloma undergoing autologous stem cell transplant
Source: Blood Cancer J. 2023 Sep 26;13(1):151. doi: 10.1038/s41408-023-00920-9 (PMC10522581; doi:10.1038/s41408-023-00920-9)
Supplement: Supplementary file 2 — Supplementary Material [file 41408_2023_920_MOESM2_ESM.docx]

| **Supplemental table 1**. Mass cytometry panels and their respective antibody targets. | | | | | | |
| --- | --- | --- | --- | --- | --- | --- |
| **Metal tag** | **Antibody target** | | **Clone** | | **Source** |  |
|  | **Lymphoid panel** | **Myeloid panel** | **Lymphoid** | **Myeloid** | **Lymphoid** | **Myeloid** |
| 89Y | CD45 | CD45 | HI30 | HI/30 | Fluidigm | Fluidigm |
| 141Pr | CD196/CCR6 | CD141 | G034E3 | 1A4 | Fluidigm | Custom |
| 142Nd | CD19 | CD19 | HIB19 | HIB19 | Fluidigm | Fluidigm |
| 143Nd | CD127/IL7Ra | CD1c | A019D5 | L161 | Fluidigm | Custom |
| 144Nd | CD38 | CD11b | HIT2 | ICRF44 | Fluidigm | Fluidigm |
| 145Nd | KLRG1 | CD40 | SA231A2 | 5C3 | Biolegend | Custom |
| 146Nd | CD69 | CD64 | FN50 | 10.1 | Biolegend | Fluidigm |
| 147Sm | CD159a/NKG2A | CD11c | Z199 | Bu15 | R&D Systems | Fluidigm |
| 148Nd | CD95/Fas | CD16 | DX2 | 3G8 | Biolegend | Fluidigm |
| 149Sm | CD194/CCR4 | CD303 | REA279 | 201A | Fluidigm | Custom |
| 150Nd | CD86 | CD86 | IT2.2 | IT2.2 | Fluidigm | Fluidigm |
| 151Eu | CD34 | CD123 | 581 | 6H6 | Biolegend | Fluidigm |
| 152Sm | TCRgd | CD36 | 11F2 | 5-271 | Fluidigm | Fluidigm |
| 153Eu | TIGIT | CD192 | MBSA43 | K036C2 | Fluidigm | Fluidigm |
| 154Sm | CX3CR1 | TIM-3 | 2A9-1 | F38-2E2 | Biolegend | Fluidigm |
| 155Gd | CD45RA | CD45RA | HI100 | HI100 | Fluidigm | Fluidigm |
| 156Gd | CD195/CCR5 | CD7 | NP-6G4 | CD7-6B7 | Fluidigm | Biolegend |
| 158Gd | CD27 | CD33 | L128 | WM53 | Fluidigm | Fluidigm |
| 159Tb | CD217/IL17RA | CD274/PD-L1 |  | 29E.2A3 | Biolegend | Fluidigm |
| 160Gd | CD28 | CD13 | CD28.2 | WM15 | Fluidigm | Fluidigm |
| 161Dy | CD279/PD1 | CD90 | EH12.2H7 | 5E10 | Biolegend | Fluidigm |
| 162Dy |  | CD66b |  | 80H3 | Biolegend | Fluidigm |
| 163Dy | CD183_CXCR3 | CD172a_b | G025H7 | SE5A5 | Fluidigm | Fluidigm |
| 164Dy | CD161 | CD15 | HP-3G10 | W6D3 | Fluidigm | Fluidigm |
| 165Ho | CD45RO | CD163 | UCHL1 | GHI/61 | Fluidigm | Fluidigm |
| 166Er | CD314/NKG2D | CX3CR1 | ON72 | 2A9-1 | Fluidigm | Custom |
| 167Er | CD197/CCR7 |  | G043H7 |  | Fluidigm |  |
| 168Er | CD8a | CD206 | SK1 | 15-2 | Fluidigm | Fluidigm |
| 169Tm | CD25/IL2R | CD32 | 2A3 | FUN-2 | Fluidigm | Fluidigm |
| 170Er | CD3 | CD3 | UCHT1 | UCHT1 | Fluidigm | Fluidigm |
| 171Yb | CD226 | CD195 | DX11 | NP-6G4 | Fluidigm | Fluidigm |
| 172Yb | CD57 | CD38 | HCD57 | HIT2 | Fluidigm | Fluidigm |
| 173Yb | HLA-DR | HLA-DR | L243 | L243 | Fluidigm | Fluidigm |
| 174Yb | CD4 | CD4 | SK3 | SK3 | Fluidigm | Fluidigm |
| 175Lu | CD184/CXCR4 | CD14 | 12G5 | M5E2 | Fluidigm | Fluidigm |
| 176Yb | CD56/NCAM | CD56 | NCAM16.2 | NCAM16.2 | Fluidigm | Fluidigm |
| 191Ir | DNA1 marker | DNA1 marker | 3G8 | 3G8 | Fluidigm | Fluidigm |
| 193Ir | DNA2 marker | DNA2 marker | FN50 | FN50 | Biolegend | Biolegend |
| 195Pt | Cisplatin | Cisplatin | Z199 | Z199 | R&D Systems | R&D systems |
| 209Bi | CD16 | CD47 | DX2 | CC2C6 | Biolegend | Fluidigm |


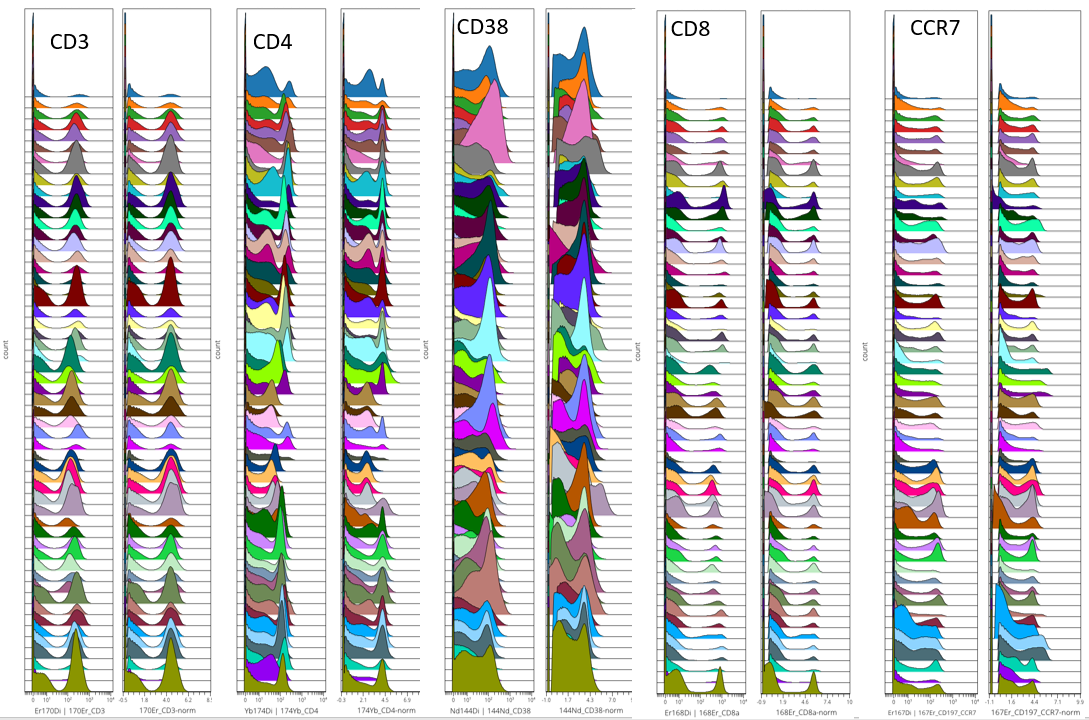


**Supplemental figure 1**. Characteristic examples of channels before (left) and after (right) normalization using fdanorm.


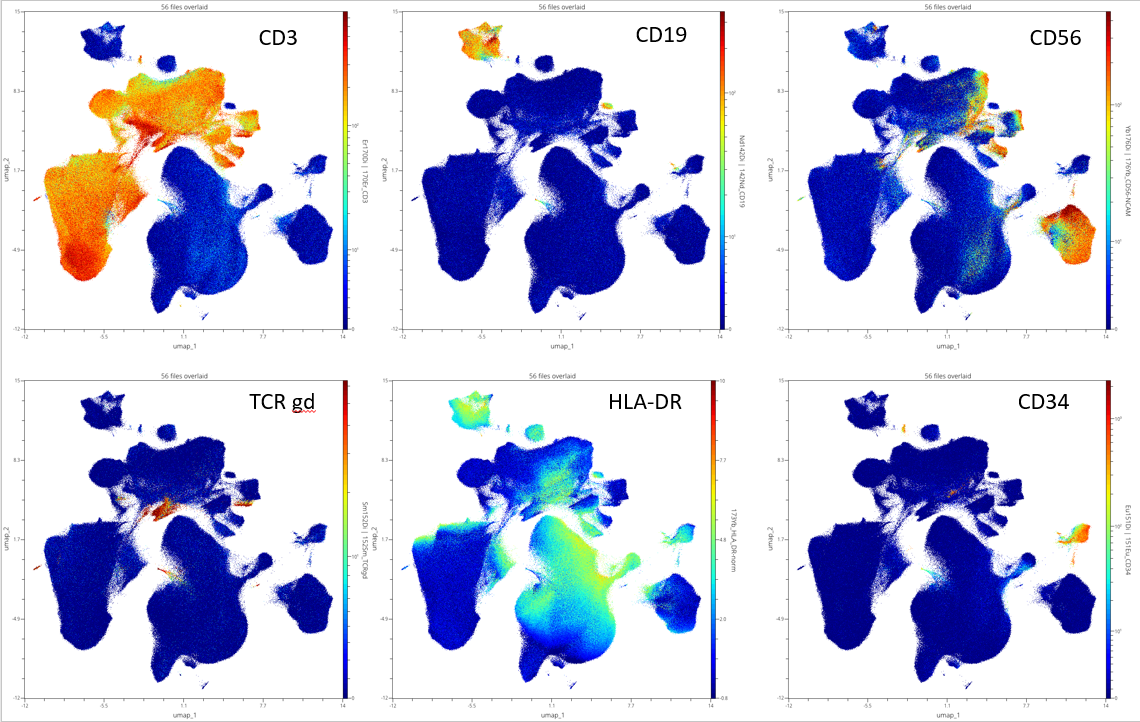


**Supplemental figure 2**. UMAP visualizing the major lineages of CD45+ cells identified in the dataset using the lymphoid panel: CD3- T cells; CD19-B Cells; CD56- T and NKT cells; TCRgd-TCR gd T cells; HLA-DR- myeloid cells, dendritic cells, and B and T cell subsets, CD34: stem cells.


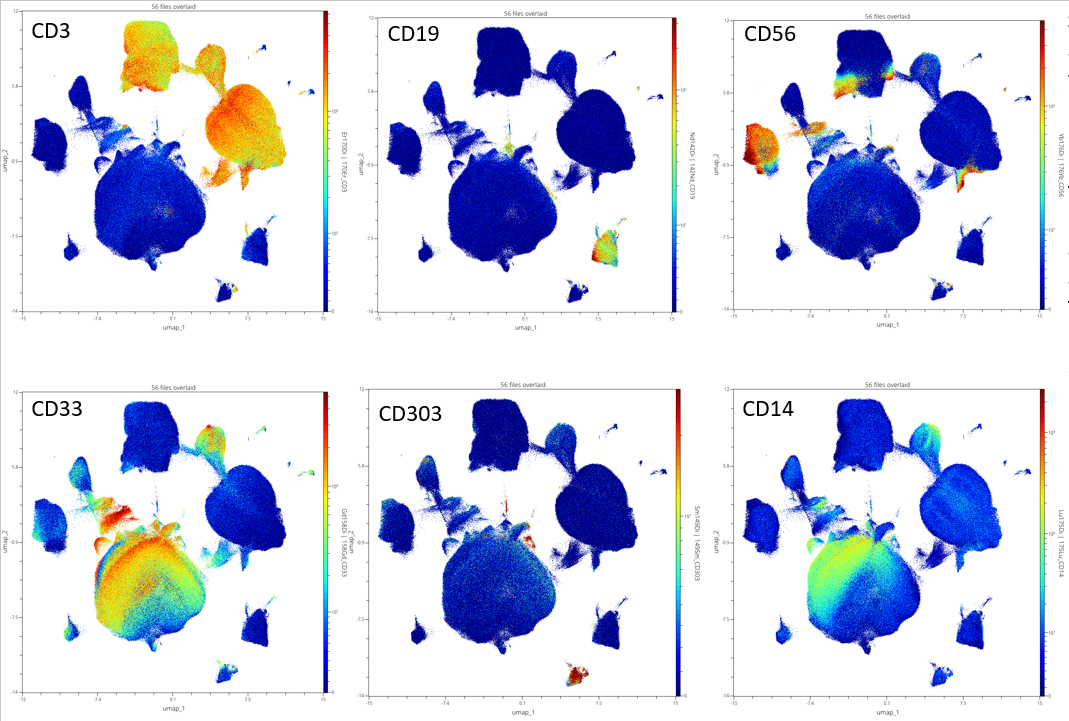


**Supplemental figure 3**. UMAP visualizing the major lineages of CD45+ cells identified in the dataset using the myeloid panel: CD3- T cells; CD19-B Cells; CD56- T and NKT cells; CD33-Myeloid cells (and some T/myeloid doublets); CD303-plasmacytoid dendritic cells, CD14-classical monocytes


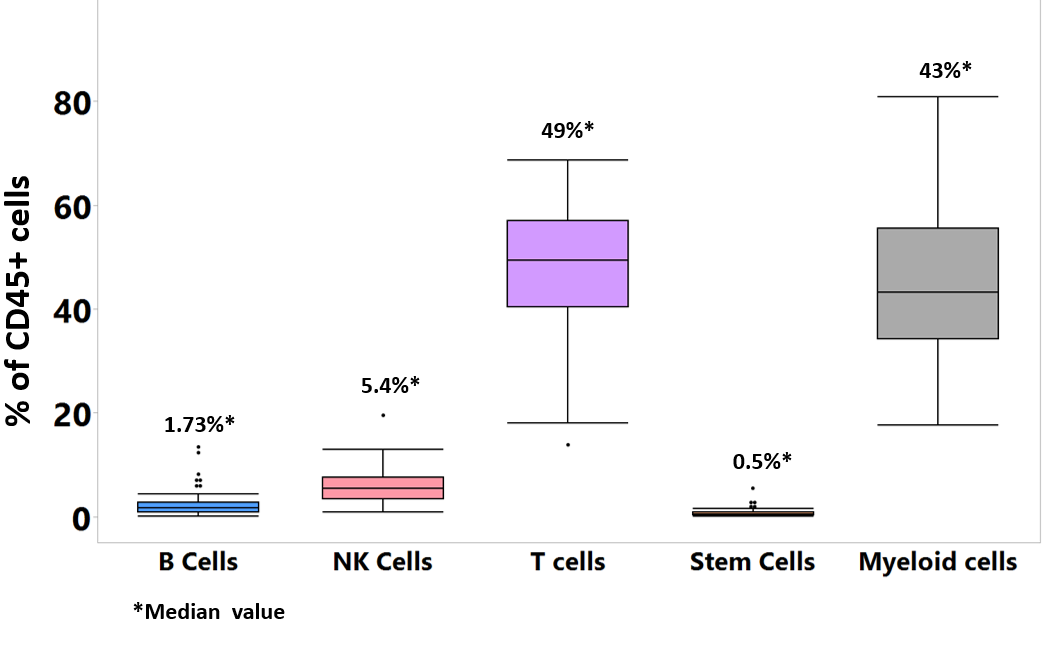


**Supplemental figure 4.** Relative proportions (% of CD45+ cells) of major cell types identified across all samples

***Correlation analyses reveal unique subsets associated with poor outcomes***

We hypothesized that the immune subsets with highly correlated frequeneices were more likely to be co-regulated. A correlation matrix and a list of all identified significant (*P*<0.05) correlations sorted by their R values are shown in ***Supplemental figure 5*** below and provided as a Supplemental spreadsheet, respectively. We defined highly correlated subsets as subsets with a Pearson’s R>0.9 (R^2^>0.81). Of note, no negative correlations with an R<-0.9 were identified. We identified some highly correlated subsets that were present in isolated patients (N=3), all of whom later developed t-MN and two of whom had short post-ASCT remission. Myeloid subsets M-14, M-21 and M-24 highly (R>0.99) correlated with each other and with the T-12 subset and were present in only one patient at a high frequency (36%, 8%, 4.7% and 3.2% of total CD45+ cells, respectively), and essentially absent from the rest (abundance <0.02% for all subsets in remaining patients). The patient was a 52-year-old male, who developed t-MN within 50 months from ASCT, while on lenalidomide maintenance. The M-21 subset was a CD33/CD15/CD66b+, HLA-DR- subset resembling granulocytic myeloid derived suppressor cells (MDSCs). Subset M-14 was C56/CD11b+ and has been described in the past in G-CSF mobilized peripheral blood cells as well as healthy donors, albeit in much lower frequency.^34^ [15526027] The M-24 subset was an atypical CD7/CD38/CD56+ subset consistent with myeloid progenitor cells. The T-12 subset was a PD-1/TIGIT+ exhausted CD8 T cell subset.

Subsets T-9 and M-13 also highly correlated (R=0.99) and were present in a 65-year-old female patient at a high frequency (16.4% and 41.8%) and essentially absent in the rest. M-13 is a PDL-1+ subset, that is known to promote T cell apoptosis^35^, and T-9 an immunosenescent (CD27/CD28/CD127-) CD8 T cell subset.^36, 37^ [29400704, 32580776] The patient developed t-MN 80 months from ASCT and MM relapsed 22 months from ASCT. Finally, subsets M-7 and M-16, described above and associated with severe ES, were present in a 71-year-old male (27.5% and 27.3%, respectively) who developed t-MN and relapsed MM 23 months post ASCT, while on lenalidomide maintenance. These data suggest the possibility of the presence of unique immunosuppressive cell subsets that may be associated with poor post-ASCT outcomes.


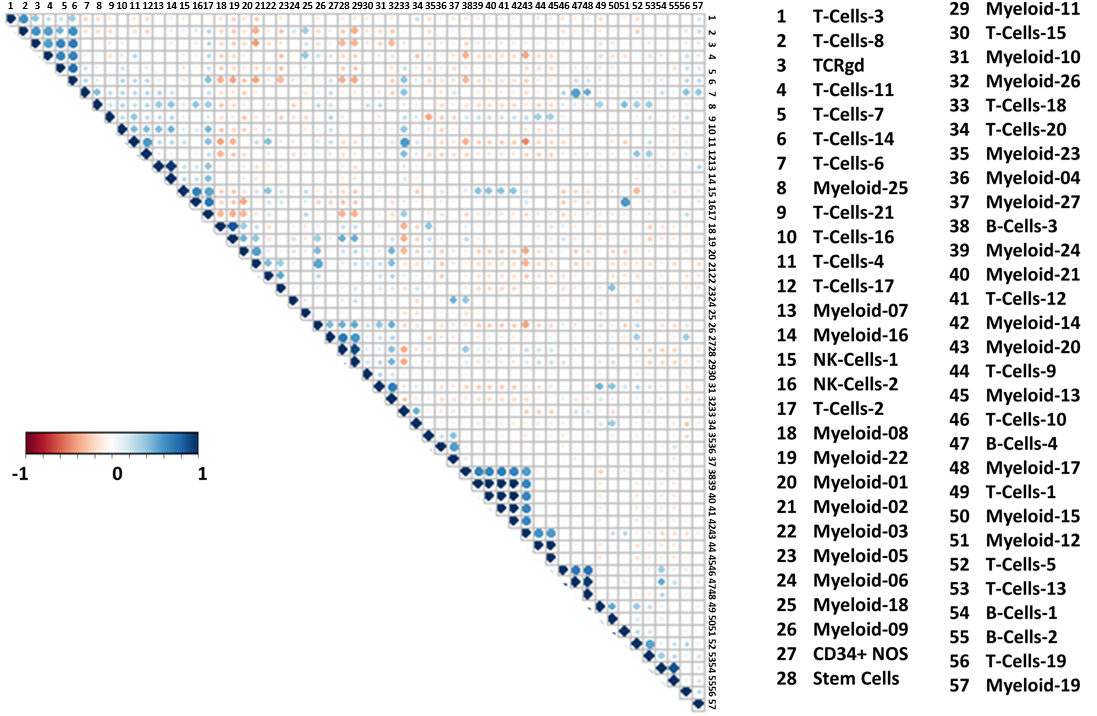


**Supplemental figure 5.** Correlation matrix of frequencies of all identified cell subsets across all samples

| **Supplemental table 2**. Differential immunophenotypic subsets in peripherally mobilized stem cell product of patients subsequently later developed t-MN compared those who did not | | | |
| --- | --- | --- | --- |
| **Immune subset** | **Developed t-MN** | **No t-MN** | ***P*-value** |
|  | **% Of CD45 cells, median (range)** | | |
| NK-cell-subcluster-7 | 0.33 (0.07-2.4) | 0.17 (0.06-0.9) | 0.006 |
| NK-cell-subcluster-15 | 0.09 (0.01-0.8) | 0.06 (0.003-0.18) | 0.001 |
| T-cells, T-5 | 1.9 (0.14-7.7) | 2.5 (0.25-9.3) | 0.02 |
| CD34+ NOS | 0.05 (0.002-0.1) | 0.07 (0.008-0.3) | 0.03 |
| T-cells, T-20 | 4.2 (1.3-9.4) | 6.24 (1.5-24.4) | 0.03 |
| Myeloid, M-20 | 0.1 (0-2.1) | 0.08 (0.01-0.5) | 0.03 |
| T-cells, T-1 | 0.2 (0.01-1.5) | 0.7 (0.05-2.7) | 0.03 |
| Myeloid, M-9 | 0.5 (0.03-1.1) | 0.7 (0.24-1.6) | 0.04 |
| NK-cell-subcluster-10 | 0.15 (0.02-1.6) | 0.1 (0-0.9) | 0.04 |
| t-MN: therapy-related myeloid neoplasm; NK – natural killer | | | |
